# Development of app-based neuropsychological tasks for screening people at mental health high risk

**DOI:** 10.1192/j.eurpsy.2025.1444

**Published:** 2025-08-26

**Authors:** M.-S. Shin, S. Um, M. Jeon, Y. Ahn

**Affiliations:** 1 Psychology, Korea University; 2 Psychiatry, Seoul National University Hospital, Seoul, Korea, Republic Of

## Abstract

**Introduction:**

Depressive and anxiety disorders have a high prevalence and pose a significant socioeconomic burden. To reduce this, the importance of early intervention through the screening of high-risk groups has been highlighted by many researchers and clinicians. However, due to the heterogeneity of symptoms and comorbidity with other mental disorders, it is challenging to identify high-risk groups of depression and anxiety disorders early on. To address this issue, we developed App-based neuropsychological tasks for screening high-risk groups and classifying subtypes of depression and anxiety disorders through services in the metaverse.

**Objectives:**

As a preliminary step, we have developed four app-based neuropsychological tasks for the early screening and subtype classification of individuals at high risk for depression and anxiety disorders, and examined the reliability and validity of the tasks.

**Methods:**

Subjects were 30 adults with mean age of 32.93(male=8, female=22). After obtaining written agreement to participate the study, smartphones with the app-based neuropsychological tasks were provided to them, and then smartphone app tasks as well as digitized self-report scales related to depression and anxiety were performed. To analyze the reliability of the tasks, Cronbach’s alpha was calculated. And correlation analyses were conducted between the scores of each task (ex; number of correct response, reaction time, number of skipping the task level) and the scores of the self-questionnaires to verify validity of the tasks. The overview of the tasks developed in this study is as follows: The catching raccoon task investigates the tendency to abandon performance in complex working memory task situations. The soccer task measures vitality and planning abilities. The risk-taking decision-making task examines the tendency to take risks. In the verbal memory task with mood induction, we investigate whether attention bias towards negative emotions, such as depression, affect the memory functions after inducing negative emotions through audiovisual stimuli.

**Results:**

The range of Cronbach’s alpha values for the task was between .71 and .92, showing that the tasks are reliable. Additionally, three out of the four tasks showed statistically significant correlations with the scores of depression or anxiety measured by self-report rating scales, confirming the validity of the tasks.

**Conclusions:**

Despite being conducted with a normal population, these results verified the reliability and validity of the tasks, suggesting the potential usefulness of the tasks for early intervention through the screening of high-risk groups with depression and anxiety disorders.

**Disclosure of Interest:**

None Declared

